# Outcomes of the modern management approach for locally advanced (T_3_–T_4_) laryngeal cancer: a retrospective cohort study

**DOI:** 10.1017/S0022215124001105

**Published:** 2024-12

**Authors:** Amarkumar Dhirajlal Rajgor, Josh Cowley, Colin Gillespie, Chang Woo Lee, James O'Hara, Muhammad Shahid Iqbal, David Winston Hamilton

**Affiliations:** 1 National Institute for Health & Care Research Doctoral Fellow in Otolaryngology, Population Health Sciences Institute, Newcastle University, Newcastle upon Tyne, UK; 2 Department of Otolaryngology – Head and Neck Surgery, The Newcastle upon Tyne Hospitals NHS Foundation Trust, Newcastle upon Tyne, UK; 3 School of Mathematics, Newcastle University, Newcastle upon Tyne, UK; 4 Department of Clinical Oncology, Northern Centre for Cancer Care, The Newcastle upon Tyne Hospitals NHS Foundation Trust, Newcastle upon Tyne, UK

**Keywords:** larynx, head and neck surgery, chemoradiotherapy, oncology

## Abstract

**Background:**

Our centre (Freeman Hospital, Newcatle Upon Tyne NHS Trust) has favoured primary surgery over chemoradiotherapy for specific advanced laryngeal cancer patients (e.g. large-volume tumours, airway compromise, significant dysphagia, T_4_ disease). This study reports the survival outcomes for a modern, high-volume head and neck centre favouring surgical management to determine whether this approach improves survival.

**Method:**

Retrospective analysis of patient data over a seven-year period from a tertiary cancer centre.

**Results:**

In total, 121 patients were identified with T_3_ (*n* = 76) or T_4_ (*n* = 45) laryngeal cancer (mean follow up 2.9 years). In the cohort treated with curative intent (*n* = 104, 86.0 per cent), the 2- and 5-year estimated disease-specific survival rates were 77.9 and 64.1 per cent. chemoradiotherapy had the highest 2-year disease-specific survival (92.5 per cent), followed by surgery with adjuvant therapy (81.8 per cent), radiotherapy alone (75 per cent) and surgery alone (72.4 per cent).

**Conclusion:**

For a centre favouring primary surgery for certain advanced laryngeal cancers, the disease-specific survival appears no higher than that found in the published literature. To enhance survival, future research should focus on precision medicine to define treatment pathways in this disease.

## Introduction

Since the 1990s head and neck cancer incidence rates have increased by a third and it is currently the eighth leading form of cancer in the UK.^1^ A large proportion of these cases occur in the larynx, accounting for 25–30 per cent of all head and neck cancer cases.^2^ If diagnosed early (stages I–II), disease-specific survival rates range from 70 to 90 per cent, but just under half of all new cases present at an advanced stage (stages III–IV) where, at best, disease-specific survival ranges from 50 to 60 per cent.^3–5^ Options for definitive treatment in advanced disease include a total laryngectomy (with or without adjuvant therapy) or non-surgical management in the form of concurrent chemoradiotherapy.

For advanced disease, there was a shift towards chemoradiotherapy following two landmark randomised controlled trials.^6,7^ However, since this shift, there have been concerns as a result of declining survival and increased recurrence rates.^8,9^ In parallel, there has also been increasing evidence describing improved survival with a surgical approach.^5,10^ This uncertainty has led to large epidemiological studies being conducted to further evaluate the role of surgery and identify patient and tumour characteristics that may be better managed with surgery. Whilst surgery is preferred for T_4_ disease, T_3_ disease can be treated with either surgery or chemoradiotherapy.^11^ Mendenhall *et al*. suggested that patients with large tumour volumes (T_3_ tumours), airway compromise requiring debulking or a tracheostomy are best managed with a surgical approach.^12^ This approach has received further support in the 2024 edition of the Head and Neck Cancer: United Kingdom National Multidisciplinary Guidelines.^13^

With this growing body of evidence, our tertiary head and neck centre (Freeman Hospital, Newcatle Upon Tyne NHS Trust) has tended to favour primary surgery over chemoradiotherapy for patients with characteristics associated with poor chemoradiotherapy outcomes, such as patients with large-volume tumours, airway compromise, significant dysphagia and/or laryngeal dysfunction and all T_4_ tumours. This study evaluates the survival outcomes of locally advanced laryngeal cancer in a unit that favours a surgical approach.

## Methods

### Patient population

Following local trust board approval, retrospective data were collated over a 7-year period (January 2012 to December 2019) from a tertiary head and neck specialist centre, including 121 patients with locally advanced laryngeal cancer (defined as T_3_ or T_4_ disease as per the tumour–node–metastasis (TNM) 7 classification system^3^). Patients without sufficient follow up or medical data were excluded. For survival (Kaplan–Meier) analysis, patients managed with palliative intent were excluded (*n* = 17*)*. However, these patients were used as a reference group in the Cox proportional hazards model.

Standard demographics were recorded, including age, sex, smoking status, TNM stage at diagnosis, anatomical site of primary malignancy and treatment modality received. Smoking status was divided into current smoker (active smoker at diagnosis), ex-smoker (stopped smoking more than eight weeks before diagnosis) and non-smoker. Additional information recorded included the requirement for an emergency tracheostomy.

### Oncological management strategies

Patients received one of five management approaches: chemoradiotherapy, surgery followed by adjuvant therapy, surgery alone, radiotherapy (RT) alone or palliation. Detailed outcomes of each treatment modality were recorded, including the time taken from diagnosis to treatment initiation, the chemotherapy agent used, the RT dosage given, details of the surgical procedure, surgical margin status and treatment complications (chemotherapy toxicity, RT toxicity and surgical complications). Radiotherapy complications were classified according to the National Cancer Institute's Common Terminology Criteria for Adverse Events.^14^

### Statistical analysis

All statistical analyses were performed using R (version 4.0.3). Overall survival, disease-specific survival and recurrence-free survival were calculated. For survival analysis, Kaplan–Meier plots were created and to identify predictors of survival the multivariate analysis using the Cox proportional hazards model was performed. Hazard ratios and the associated 95 per cent confidence levels (CIs) were reported.

## Results

### Baseline demographics

The study population included 121 patients with T_3_ (*n* = 76) or T_4_ (*n* = 45) laryngeal cancer with a mean follow up of 2.9 years. There were 90 males and 31 females. The mean age was 66 and the majority of patients were either smokers (*n* = 54, 44.6 per cent) or ex-smokers (*n* = 58, 47.9 per cent). The majority of patients had glottic and/or transglottic disease (*n* = 71, 58.7 per cent) followed by supraglottic (*n* = 47, 38.8 per cent) and subglottic disease (*n* = 3, 2.5 per cent). At the time of diagnosis, 54 patients (44.6 per cent) had nodal disease and 3 patients had distant metastases. Additionally, 15 (12.5 per cent) patients presented with stridor and thus needed emergency tracheostomies. Additional information is presented in Table 1, which also delineates the cohorts managed with curative and palliative intent individually.

**Table 1.** Patient demographics and tumour traits of the advanced laryngeal cancer population

Demographic and tumour traits	Entire cohort, *n* (%)	Curative intent cohort, *n* (%)	Palliative intent cohort, *n* (%)
Sample size	121	104	17
Gender			
– Male	90 (74.4)	78 (75.0)	12 (70.6)
– Female	31 (25.6)	26 (25.0)	5 (29.4)
Age (years)	65.98 ± 11.95	65.18 ± 11.74	70.88 ± 12.44
Smoking status			
– Non-smoker	9 (7.4)	8 (7.7)	1 (5.9)
– Ex-smoker	58 (47.9)	49 (47.1)	5 (29.4)
– Current smoker	54 (44.6)	47 (45.2)	11 (64.7)
TNM status (TNM 7)			
– T stage			
– T*_3_*	76 (62.8)	71 (68.2)	5 (29.4)
– T*_4_*	45 (37.2)	33 (31.7)	12 (70.6)
– N stage			
– N*_0_*	67 (55.4)	59 (56.7)	8 (47.1)
– N*_1_*	17 (14.0)	16 (15.4)	1 (5.9)
– N*_2a_*	3 (2.5)	2 (1.9)	1 (5.9)
– N*_2b_*	16 (13.2)	12 (11.5)	4 (23.5)
– N*_2c_*	15 (12.4)	13 (12.5)	2 (11.8)
– N*_3a_*	3 (2.5)	2 (1.9)	1 (5.9)
– N*_3b_*	0 (0.0)	0 (0.0)	0 (0.0)
– M stage			
– M*_0_*	118 (97.5)	104 (100)	14 (82.4)
– M*_1_*	3 (2.5)	0 (0)	3 (17.6)
Tumour subsite			
– Subglottic	3 (2.5)	2 (1.9)	1 (5.9)
– Transglottic and/or glottis	71 (58.7)	62 (59.6)	9 (52.9)
– Supraglottis	47 (38.8)	40 (38.5)	7 (41.2)
Required emergency tracheostomy	15 (12.4)	10 (9.6)	5 (29.4)

TNM = tumour–node–metastasis

### Treatment approach

In the entire cohort, 104 patients (86.0 per cent) were treated with curative intent, of which 14 patients (11.6 per cent) received RT, 40 (33.1 per cent) received chemoradiotherapy, 19 (15.7 per cent) underwent surgery alone and 31 (25.6 per cent) underwent surgery with adjuvant treatment (Table 2). Although the majority of patients with T_3_ disease underwent chemoradiotherapy (48 per cent), almost a third (31 per cent) were treated surgically. Additionally, 85 per cent of patients with T_4_ disease underwent surgery (Supplementary Appendix 1).

**Table 2.** Treatment approach and key outcomes in patients managed with curative intent

Treatment approach and outcome	*n* (%)
Primary treatment approach (*n* (%))	
– Radiotherapy	14 (11.6)
– Chemoradiotherapy	40 (33.1)
– Surgery	19 (15.7)
– Surgery with adjuvant treatment	31 (25.6)
Time from diagnosis to treatment initiation (presented as median (IQR))	
– Radiotherapy	42 days (32)
– Chemoradiotherapy	41 days (20)
– Surgery	22 days (13)
– Surgery with adjuvant treatment	21 days (18)
Radiotherapy (*n* (%))	
– 65 Gy in 30 fractions	14 (100)
Radiotherapy toxicity (*n* (%))	
– Greater than grade 3 severity	7 (50.0)
Chemoradiotherapy (*n* (%))	
– Chemotherapy agent	
– Cisplatin	32 (80.0)
– Cetuximab	8 (20.0)
– Chemotherapy toxicity	
– Yes	12 (30.0)
– No	28 (70.0)
Surgery alone	
– Total laryngectomy	16
- Pharyngolaryngectomy	3
– Neck dissection (*n* (%))	
– None	2 (10.5%)
– Ipsilateral ND	2 (10.5%)
– Bilateral ND	15 (78.9)
– Margins (*n* (%))	
– Positive	1 (5.3)
– Negative	17 (89.5)
– Narrowly excised	1 (5.3)
Surgery with adjuvant therapy	
– Total laryngectomy	21
Pharyngolaryngectomy	10
– Neck dissection (*n* (%))	
– None	1 (3.2)
– Unilateral ND	1 (3.2)
– Bilateral ND	29 (93.5)
– Margins (*n* (%))	
– Positive	1 (3.2)
– Negative	27 (87.1)
– Narrowly excised	3 (9.7)
– Adjuvant treatment (*n* (%))	
– Chemoradiotherapy	6 (19.4)
– Radiotherapy	25 (80.6)
Recurrence (*n* (%))	
– Overall recurrence rate	27.2%
– Recurrence based on primary treatment	
– Radiotherapy	7 (50)
– Chemoradiotherapy	7 (17.5)
– Surgery	6 (31.6)
– Surgery with adjuvant treatment	8 (25.8)
– Location of recurrence	
– Local	20 (71.4)
– Laryngeal	18 (64.3)
– Stomal	2 (2.0)
– Regional	4 (14.3)
– Cervical	4 (3.8)
– Distant	11 (39.3)
– Bone	2 (1.9)
– Brain	1 (1.0)
– Lung	8 (7.7)
– Liver	5 (4.8)
– Peritoneal	2 (1.9)
– Treatment provided for recurrence	
– Radiotherapy	5 (4.9)
– Chemoradiotherapy	1 (1.0)
– Surgery	8 (7.8)
– Surgery with adjuvant treatment	2 (1.9)
– Palliation	11 (10.7)
Mortality (%)	
– Overall mortality rate	51.2
– Mortality based on treatment regimen (%)	
– Radiotherapy	78.6
– Chemoradiotherapy	27.5
– Surgery	68.4
– Surgery with adjuvant treatment	38.7

IQR = interquartile range; ND = neck dissection

For patients undergoing primary RT (*n* = 14), all patients received 65 Gy in 30 fractions, with 50 per cent developing grade 3 or greater complications. In the chemoradiotherapy cohort, 32 patients (80 per cent) received cisplatin-based chemotherapy, whilst 8 (20 per cent) received cetuximab. In the surgery alone group (*n* = 19), all patients underwent a laryngectomy with or without a pharyngectomy, of which 17 patients (89.5 per cent) had a neck dissection (ipsilateral *n* = 2, bilateral *n* = 15) and 17 had completely negative margins. In the surgery with adjuvant therapy cohort (*n* = 31), 10 patients underwent a laryngopharyngectomy and 30 (96.7 per cent) underwent a neck dissection (ipsilateral *n* = 1, bilateral *n* = 29). In addition, 27 of 31 patients had completely negative margins. These results and further details are provided in Table 2 and Supplementary Appendix 1.

### Recurrence and mortality rates

The overall recurrence and mortality rates were 27.2 and 51.2, per cent respectively. Recurrence locations encompassed local (e.g. laryngeal and stomal), regional (cervical lymph nodes) and distant (e.g. lung, liver and bone) sites. Chemoradiotherapy had the lowest recurrence rate (17.5 per cent) followed by surgery with adjuvant therapy (25.8 per cent), surgery alone (31.6 per cent) and finally RT alone (50 per cent). Chemoradiotherapy also had the lowest overall mortality rate (27.5 per cent), followed by surgery with adjuvant therapy (38.7 per cent), surgery alone (68.4 per cent) and RT (78.6 per cent). Further details are provided in Table 2.

### Survival outcomes

**Figure 1.**
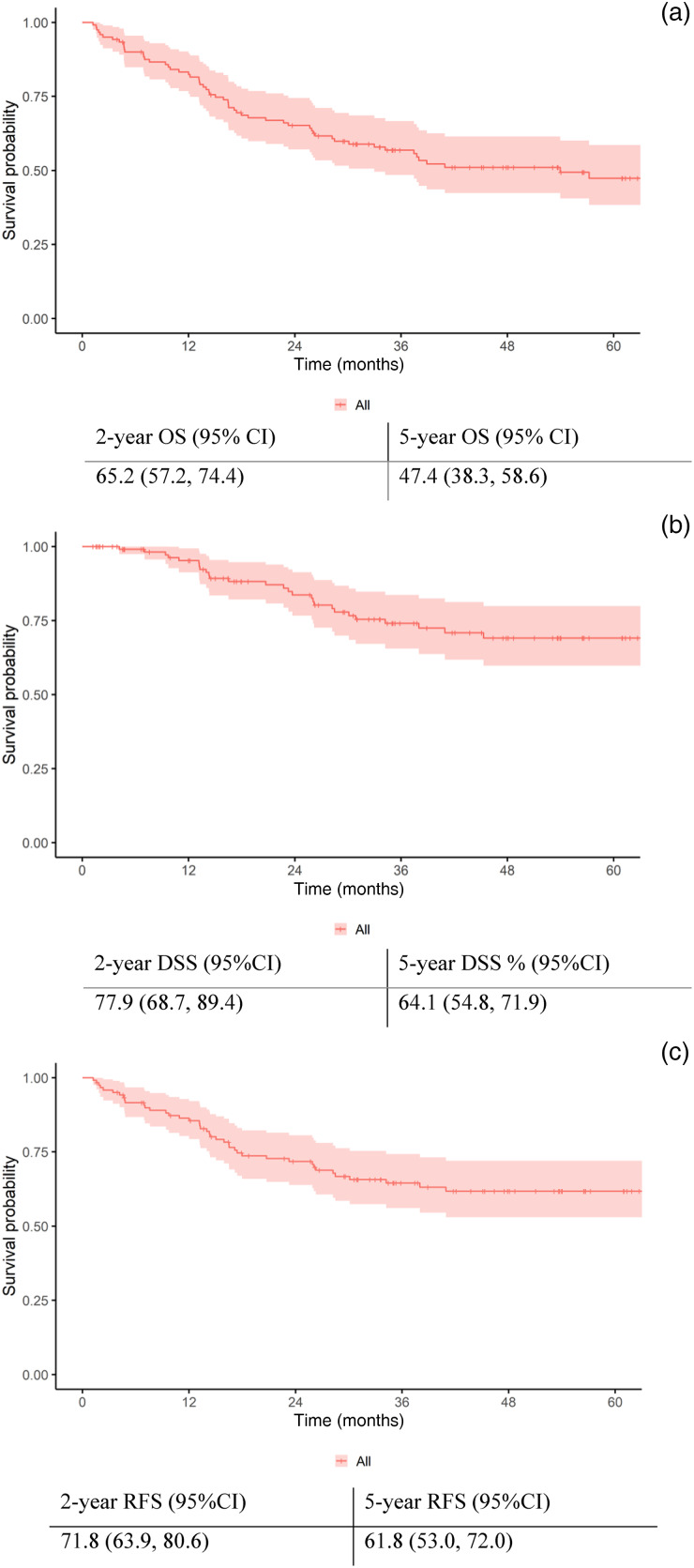
Kaplan–Meier survival curves for the cohort managed with curative intent: (a) overall survival, (b) disease-specific survival and (c) recurrence. OS = overall survival; CI = confidence interval; DSS = disease-specific survival; RFS = recurrence-free survival.

The survival outcomes for our cohort treated with curative intent are illustrated in Figure 1. The 5-year estimated overall survival, disease-specific survival and recurrence-free survival rates were 47.4 per cent, 64.1 per cent and 61.8 per cent, respectively.

**Figure 2.**
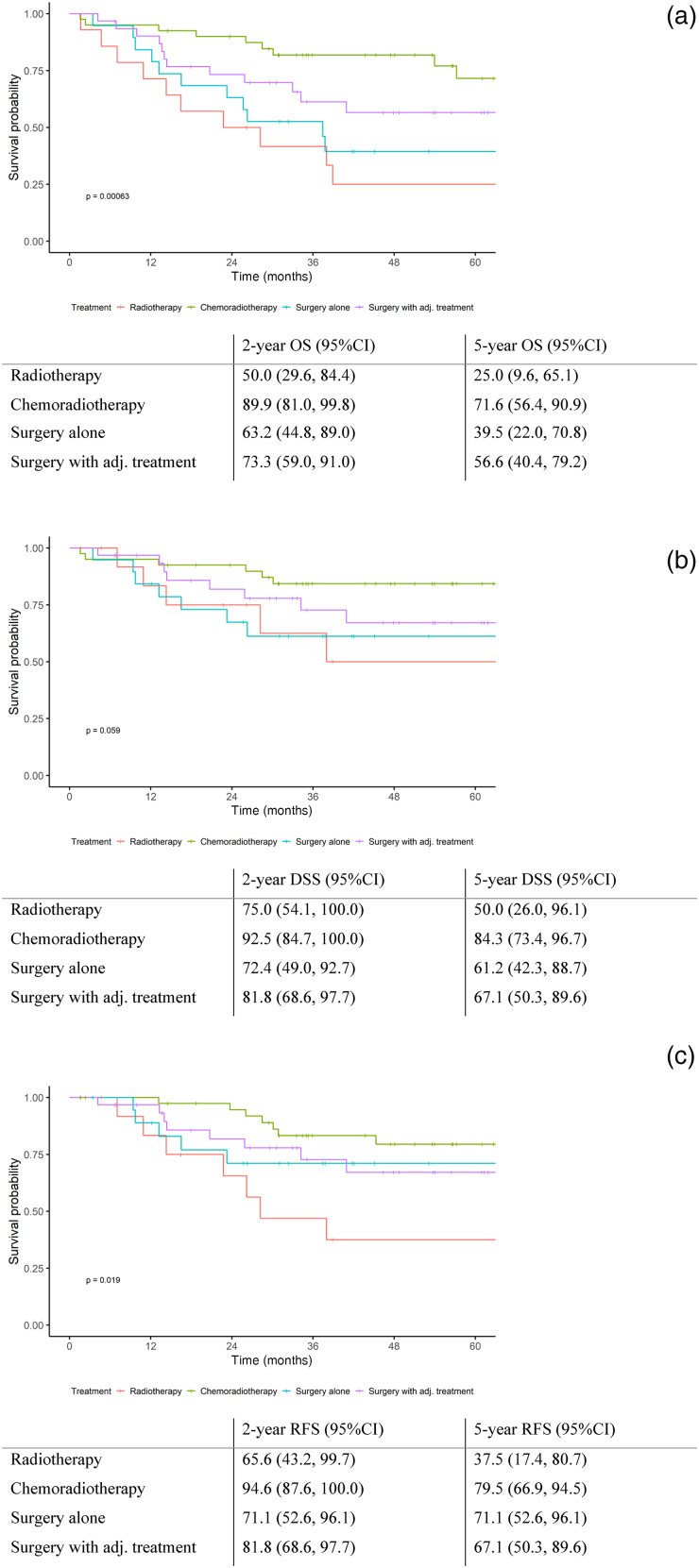
Kaplan–Meier survival curves based on treatment modality: (a) overall survival, (b) disease-specific survival and (c) recurrence. adj. = adjuvant treatment; OS = overall survival; CI = confidence interval; DSS = disease-specific survival; RFS = recurrence-free survival.

The chemoradiotherapy group had the highest 2- and 5-year estimated overall survival rates, followed by patients receiving surgery with adjuvant therapy, surgery alone and RT (Figure 2A). The chemoradiotherapy group had the highest wo-year estimated disease-specific survival, followed by surgery with adjuvant therapy, RT alone and surgery alone (Figure 2B). Following a similar trend, the chemoradiotherapy group had a the highest two-year recurrence-free survival, followed by surgery with adjuvant treatment, surgery alone and RT alone (Figure 2C). Further details with estimated survival rates are provided in Figure 2.

### Predictors of overall survival and disease-specific survival

Multivariate analysis demonstrated that age, tumour subsite and treatment modality significantly affected overall survival. These factors also remained significant for disease-specific survival. Respective hazard ratios, 95 per cent CIs and *p* values are detailed in Table 3. Gender, smoking status, nodal status and tumour stage had no statistically significant impact on survival.

**Table 3.** Multivariate analysis demonstrating factors predictive of survival

Variable	Overall survival model		Disease-specific survival model	
	Hazard ratio (95% CI)	*p*	Hazard ratio (95% CI)	*p*
Age	0.47 (0.17, 7.8)	0.002*	0.04 (0.01, 0.08)	0.021*
Gender				
– Male	Ref		Ref	
– Female	−0.18 (−0.57, 55.8)	0.54	−0.46 (−0.76, 0.22)	0.137
Smoking status				
– Non-smoker	Ref		Ref	
– Ex-smoker	−0.50 (−0.87, 0.95)	0.315	−0.59 (−0.91, 0.90)	0.255
– Current smoker	−0.20 (−0.80, 2.21)	0.749	−0.72 (−0.94, 0.24)	0.094
Nodal status				
– N_0_	Ref		Ref	
– N_1+_	0.40 (−0.24, 1.57)	0.287	0.86 (−0.12, 2.93)	0.102
Tumour stage				
– T*_3_*	Ref		Ref	
– T*_4_*	0.58 (−0.19, 2.12)	0.179	0.55 (−0.34, 2.66)	0.313
Tumour subsite				
– Transglottic and/or glottic	Ref		Ref	
– Subglottis	−1.0 (−1.0, Inf)	0.996	−1.0 (−1.0, Inf)	0.997
– Supraglottis	1.31 (0.14, 3.67)	0.021*	1.90 (0.21, 5.94)	0.017*
Treatment modality				
– Palliation	Ref		Ref	
– Chemoradiotherapy	−0.90 (−0.96, −0.72)	<0.001*	−0.95 (−0.98, −0.83)	<0.001*
– Radiotherapy	−0.67 (−0.87, −0.18)	0.017*	−0.81 (−0.94, −0.39)	0.005*
– Surgery alone	−0.72 (−0.90, −0.27)	0.01*	−0.85 (−0.95, −0.51)	0.001*
– Surgery with adjuvant therapy	−0.84 (−0.93, −0.61)	<0.001*	−0.91 (−0.97, −0.74)	<0.001*

CI = confidence interval; Ref = Reference Category ; Inf = Infinity

## Discussion

In our retrospective study, for patients managed with curative intent the 2- and 5-year disease-specific survival rates were 77.9 and 64.1 per cent, respectively. Patients receiving concurrent chemoradiotherapy had the greatest survival outcome followed by surgery with adjuvant therapy, surgery alone and RT alone.

The management approach for locally advanced laryngeal cancer has been heavily debated over the last three decades. In 1991, the Veterans Affair study demonstrated it was possible to treat locally advanced laryngeal cancer with a non-surgical approach (chemoradiotherapy) without compromising survival.^7^ Subsequently, in 2003, the Radiation Therapy Oncology Group (RTOG 91-11 study further solidified the value of chemoradiotherapy.^8^ This resulted in a treatment paradigm shift towards chemoradiotherapy for advanced disease. However, since this implementation, survival rates have declined and there has been increasing evidence of superior survival outcomes with a surgical approach.^5,9^ This has resulted in further research evaluating the role of surgery in advanced laryngeal cancer.

A recent literature review published by Mendenhall *et al*. emphasised that although low-volume T_3_–T_4_ tumours can be treated with chemoradiotherapy, higher-volume tumours, particularly those with airway compromise, should be treated with primary surgery and adjuvant therapy.^12^ This approach is further affirmed in the 2024 Head and Neck Cancer: United Kingdom National Multidisciplinary Guidelines, and therefore it has been adopted by our tertiary centre. Thus, patients with large-volume T_3_ disease as well as all T_4_ tumours have tended to undergo surgery. This is evident in our study population, with approximately a third (31 per cent) of T_3_ disease and 85 per cent of T_4_ disease being treated with surgery. Of these patients, 12.5 per cent presented with stridor and required urgent intervention. Comparatively, in the University of Michigan experience, only 17 per cent of T_3_ disease and 27 per cent of T_4_ disease patients received primary surgery.^10^ Thus, our study provides survival outcomes in an academic centre that has accounted for the evolving evidence.

Unfortunately, despite adopting this evidence-based approach, it appears our survival rates are no better than the published literature.^5,15–18^ Even going as far back as 1999, the 5-year survival rates are similar.^19,20^ When compared with a study conducted in Australia focusing on advanced glottic disease, our cohort exhibited similar 5-year disease-specific survival rates (64.1 *vs* 63.8 per cent). Additionally, the 5-year disease-specific survival rates for patients undergoing surgery were marginally lower in our cohort (67.1 *vs* 71.2 per cent). In comparison to Wolf *et al*., our cohort's 5-year disease-specific survival rate was lower (64.1 *vs* 78 per cent).^10^ Our 5-year disease-specific survival rate for patients undergoing surgery was also lower (67.1 *vs* 91 per cent).

On the other hand, the 5-year disease-specific survival rate for patients receiving primary chemoradiotherapy was seemingly higher than the Michigan experience (84.3 *vs* 66 per cent).^10^ These results may be a consequence of surgery being offered to more advanced disease as well as our specialist multidisciplinary team's ability to appropriately select patients with laryngeal disease amenable to chemoradiotherapy. These results may reflect different patient populations, but they may also indicate that our pendulum for advocating surgery may have shifted too far away from non-surgical management.

As it stands, survival has not improved in advanced laryngeal cancer treatment for over 30 years. For advanced cases, a significant challenge faced by both clinicians and patients is deciding between chemoradiotherapy or a laryngectomy. Both treatment options are life-changing and can have a significant impact on a patient's quality of life. These treatments can result in swallowing difficulties, require a tracheostoma, impact voice and negatively impact a patient's psychosocial wellbeing.^21^ Despite this, clinicians have limited information to aid treatment decision-making. Favouring either chemoradiotherapy or surgery for advanced disease has the potential to compromise survival outcomes.

Our excellent chemoradiotherapy 5-year disease-specific survival rate of 84.3 per cent clearly demonstrates that appropriately selected patients can do very well, but the difficulty lies in how to best identify them. One particular study conducted by Wolf *et al*. investigated the potential for using a single cycle of neoadjuvant chemotherapy to select patients with advanced disease for either laryngectomy or concurrent chemoradiation.^10^ They demonstrated that disease-specific survival significantly improved in the patients selected for chemoradiation using the single-cycle treatment compared with those without neoadjuvant selection (hazard ratio 0.48, *p* = 0.005).

- Concurrent chemoradiotherapy has been favoured for organ preservation, but there has been some evidence supporting the role of surgery in the management of advanced laryngeal cancer with specific tumour characteristics for better oncological control
- In our experience, patients receiving concurrent chemoradiotherapy had the greatest survival outcomes followed by surgery with adjuvant therapy, surgery alone and radiotherapy alone
- Adopting a modern approach to managing advanced laryngeal cancer and favouring a surgical approach for certain tumours has no superior benefit for survival
- To improve advanced laryngeal cancer survival, research needs to move away from identifying treatment superiority and focus on precision medicine to define treatment pathways in this disease

To further laryngeal cancer research we need to adopt such approaches and move towards personalised medicine and providing tumour-specific treatment plans. The creation of decision-making models incorporating clinicopathological features (e.g. demographics, risk factors for disease, histopathological features) and even novel artificial intelligence techniques such as radiomics could be key in aiding prognostication.^22^ Through such models, we could potentially deliver chemoradiotherapy to patients likely to respond, operate on those who may not and palliate those patients unlikely to benefit from either treatment rather than subjecting them to extensive radical treatment. The 2024 Head and Neck Cancer: United Kingdom National Multidisciplinary Guidelines also supports this rationale and emphasises the importance of innovative research endeavours, such as radio-genomics and chemo-selection protocols, aimed at enhancing patient selection for primary treatment. This, in turn, lays the groundwork for personalised medicine.

Laryngeal cancer survivorship could also improve with the discovery and implementation of novel therapeutic agents. However, over the last two decades there has been very limited progress in this field. There are laboratory studies evaluating novel targets for laryngeal cancer such as the 5-hydroxytryptamine receptor 7, but further extensive analysis is required prior to translation to clinical care.^23^

### Limitations

The limitations of this study include its retrospective nature and the relatively small sample population This makes drawing concrete conclusions challenging. Nonetheless, the sample population is larger than similar published studies on advanced disease. Additionally, the effects of co-morbidities and variation in the time to treatment initiation on survival were not explored.

## Conclusion

In a centre with a treatment philosophy that has implemented evidence-based medicine and has favoured primary surgery for certain advanced laryngeal cancers, the disease-specific survival remains no higher than that found in the current literature. Our results do not support the large epidemiological studies suggesting surgery is associated with greater oncological control. The disease-specific survival following chemoradiotherapy is encouraging and it is likely that more of our patients may have benefitted from this treatment.

However, to enhance survival, future research needs to move away from identifying treatment superiority (between chemoradiotherapy and surgery) and focus on precision medicine and novel therapeutics to provide patients with individualised tumour-specific treatment plans.

## Supporting information

Rajgor et al. supplementary material Rajgor et al. supplementary material

## Supplementary material

The supplementary material for this article can be found at https://doi.org/10.1017/S0022215124001105.
