# Supplementary material for: Outcomes of the modern management approach for locally advanced (T3–T4) laryngeal cancer: a retrospective cohort study
Source: J Laryngol Otol. 2024 Oct 21;138(12):1154–60. doi: 10.1017/S0022215124001105 (PMC11735189; doi:10.1017/S0022215124001105)
Supplement: Rajgor et al. supplementary material [file S0022215124001105sup001.docx]

**Appendix 1:** Treatment provided based on Tumour stage & Nodal status

|  | Radiotherapy | CRT | Surgery | Surgery with adjuvant therapy |
| --- | --- | --- | --- | --- |
| Tumour Stage  T3 | 12 | 37 | 13 | 9 |
| T4 | 2 | 3 | 6 | 22 |
| Nodal Stage |  |  |  |  |
| N0 | 10 | 27 | 11 | 11 |
| N1+ | 4 | 13 | 8 | 20 |
